# The Soluble Receptor for Vitamin B_12_ Uptake (sCD320) Increases during Pregnancy and Occurs in Higher Concentration in Urine than in Serum

**DOI:** 10.1371/journal.pone.0073110

**Published:** 2013-08-27

**Authors:** Omar Abuyaman, Birgitte H. Andreasen, Camilla Kronborg, Erik Vittinghus, Ebba Nexo

**Affiliations:** 1 Department of Clinical Biochemistry, Aarhus University Hospital, Aarhus, Denmark; 2 Department of Clinical Biochemistry, Randers County Hospital, Randers, Denmark; 3 Department of Oncology, Aarhus University Hospital, Aarhus, Denmark; Gentofte University Hospital, Denmark

## Abstract

**Background:**

Cellular uptake of vitamin B_12_ (B12) demands binding of the vitamin to transcobalamin (TC) and recognition of TC-B12 (holoTC) by the receptor CD320, a receptor expressed in high quantities on human placenta. We have identified a soluble form of CD320 (sCD320) in serum and here we present data on the occurrence of this soluble receptor in both serum and urine during pregnancy.

**Methods:**

We examined serum from twenty-seven pregnant women (cohort 1) at gestational weeks 13, 24 and 36 and serum and urine samples from forty pregnant women (cohort 2) tested up to 8 times during gestational weeks 17-41. sCD320, holoTC, total TC and complex formation between holoTC and sCD320 were measured by in-house ELISA methods, while creatinine was measured on the automatic platform Cobas 6000. Size exclusion chromatography was performed on a Superdex 200 column.

**Results:**

Median (range) of serum sCD320 increased from 125 (87-839) pmol/L (week 15) to reach a peak value of 199 (72-672) pmol/L (week 35) then dropped back to its baseline level just before birth (week 40). Around one third of sCD320 was precipitated with holoTC at all-time points studied. The urinary concentration of sCD320 was around two fold higher than in serum. Urinary sCD320/creatinine ratio correlated with serum sCD320 and reached a peak median level of 53 (30–101) pmol/mmol creatinine (week 35). sCD320 present in serum and urine showed the same elution pattern upon size exclusion chromatography.

**Conclusion:**

We report for the first time that sCD320 is present in urine and in a higher concentration than in serum and that serum and urine sCD320 increase during pregnancy. The high urinary concentration and the strong correlation between urinary and serum sCD320 suggests that sCD320 is filtered in the kidney.

## Introduction

Vitamin B_12_ (B12) is essential for normal fetal development [1,2]. The mother absorbs ingested B12 through a gastric intrinsic factor-mediated uptake in the ileal enterocytes [3]. After absorption, B12 is bound to transcobalamin (TC). TC circulates in plasma partly saturated with B12 (holoTC) and partly in its free form (apoTC) [3]. Due to its relatively low molecular mass of around 43-kDa [4] the molecule is filtered in the kidney, but reabsorbed in the proximal tubules [5,6]. HoloTC is essential for the receptor mediated cellular uptake of B12 [3].

During pregnancy the absorption of B12 ensures the B12 status not only for the mother but also for the fetus. While holoTC remains unchanged, total TC and B12 show declining levels in late pregnancy [2,7,8].

A holoTC binding receptor was recently purified from human placenta, and launched as the receptor mediating the uptake of holoTC in most cells [9]. This receptor, named CD320, binds holoTC and only to a much lesser degree apoTC. CD320 belongs to the low-density lipoprotein receptor family. Its 282-amino acid sequence includes a signal peptide of 31 residues, an extracellular domain of 198 residues, a transmembrane region of 21 residues, and a cytoplasmic domain of 32 residues. The binding of CD320 to holoTC does not require the cytoplasmic domain or its orientation in the plasma membrane. The extracellular domain (sCD320) still binds holoTC with high affinity and specificity [10]. sCD320 is heavily glycosylated and behave as a 58-kDa molecule upon sodium dodecyl sulfate-polyacrylamide gel electrophoresis [9,10].

We recently identified soluble CD320 (sCD320) in human serum, and successfully developed an ELISA method for its measurement [10]. Further, we showed a positive correlation between circulating sCD320 and both total B12 and holoTC [10,11]. We did not find any obvious clinical associations to serum sCD320 levels, nor did we find evidence to suggest sCD320 as a novel biomarker for B12 deficiency [11]. The function of the recently discovered sCD320, the mechanism and regulation of its release remains unknown.

In this study, we present data to show that both serum and urinary levels of sCD320 increase with gestational weeks but decline towards birth, and we present data supporting an unusual kidney handling of the sCD320 glycoprotein. 

## Materials and Methods

### Participants’ characteristics and study design

Sixty-seven pregnant Danish women from two longitudinal cohorts were included in this cross sectional study. The gestational age was defined based on the last menstrual date and the ultrasound examination. All women had healthy uncomplicated pregnancies and no chronic systemic diseases. Age, parity, sampling week, sample type, recruitment venue and date are described in Table 1. Though both cohorts represent longitudinal studies, our choice was to treat the data as a cross sectional study, as no systematic differences were observed in the results obtained from the two cohorts. The samples were divided according to the following six gestational intervals (in weeks): 12-17(15 +2/-3), 18-22(20±2), 23-27(25±2), 28-32(30±2), 33-37(35±2), and 38-42(40±2).

**Table 1:** Participants’ and samples characteristics.

	**Cohort 1(n=27)**	**Cohort 2(n=40)**
**Age mean (range)**	30 (23–39)	29 (22-35)
**Parity mean (range)**	1.6 (1-3)	1.6 (1-3)
**Twins**	0	0
**Sampling**	gestational weeks 13 (-1/+3), 24 (±1) and 36 (±1)	up to 8 times from gestational week 17 to 40
**Sample type**	Serum(non-fasting)	Serum and urine(non-fasting)
**Recruitment Venue**	Aarhus University Hospital, Denmark	Randers Regional Hospital, Denmark
**Recruitment date**	October 2008 to February 2009	October 2003 to February 2005

Basic characteristics of the study population of pregnant women along with sampling information. Age and parity are displayed as means (ranges).

The study was approved by the Central Denmark Region Ethics Committee (project numbers 20080090 and 20010153) and was performed within the confines of the Helsinki Declaration. All participants gave their written informed consent before inclusion in the study.

### Biochemical analyses

sCD320 was analyzed by an in-house sandwich ELISA (total imprecision of 4.0-8.0% and an intra-assay imprecision of 3.5-4.3%) [10], but standardized employing recombinant sCD320 (R&D Systems, Denmark) as a calibrator with a molarity calculated based on manufacture provided information. The conversion factor between the previously employed arb.u. and pmol/L is: 1 arb.u. ^≈^ 5 pmol/L. sCD320-holoTC complex was estimated by measuring serum sample for sCD320 before and after exposure to anti-TC coated magnetic beads as previously described [12]. Total TC was measured by an in-house sandwich ELISA (total imprecision of 4%–6% and an intra-assay imprecision of 3%) [13]. HoloTC was measured by the TC-ELISA after removal of the apoTC with B12-coated beads (total imprecision of 8% and an intra-assay imprecision of 4%) [14,15]. Creatinine was assayed on the Cobas 6000 automatic platform (Roche, Japan) (total imprecision of 1.2% and an intra-assay imprecision of 1.1%).

Size exclusion chromatography was performed on a Superdex 200 HR 10/30 column (GE Healthcare, Europe, Broendby, Denmark) attached to a Dionex ICS-3000 chromatography system (Dionex Corporation, Sunnyvale, CA, USA). Injected urine and serum samples (350 µL each) eluted with a flow rate of 400 μL/min for 70 min employing a Tris buffer (0.1 mol/L) (Sigma Aldrich, Broendby, Denmark), 1 mol/L NaCl (Merck, Denmark, Hellerup, Denmark), 0.5 g/L bovine albumin (Sigma Aldrich), 0.2 g/L sodium-azide, pH 8.0 (Merck, Denmark). The eluted fractions were measured for sCD320 content as indicated above. Blue Dextran (Sigma-Aldrich, Broendby, Denmark) and 22Na (GE Healthcare, Broendby, Denmark) was used for determination of void volume (V_0_) and total volume (V_t_), respectively. Stokes radius was calculated as described [16] using the stokes radius of holoTC (2.55 nm) as internal standard [17].

### Statistical analysis

B12 related parameters did not follow normal distribution (using Kolmogorov-Smirnov and Shapiro-Wilk normality tests). Thus, non-parametric statistical tests were used and levels of B12 related parameters were reported as medians with 95% confidence interval. Mann-Whitney U-test and Wilcoxon signed rank test were applied for testing the difference between median levels. Spearman’s rank correlation was used to correlate B12 related parameters. Statistical analyses were performed using SPSS statistical computer software for WINDOWS (version 20, IBM Inc., New York, USA, www.ibm.com/ software / analytics /spss/). 

## Results

We present data on the occurrence of sCD320 in two cohorts of pregnant women. The characteristics of the two populations are indicated in table 1.

We employed a previously established assay for measurement of sCD320 [10]. However in order to allow for a molar determination of sCD320 we standardized the assay employing a commercially available human recombinant CD320.

**Figure 1:**
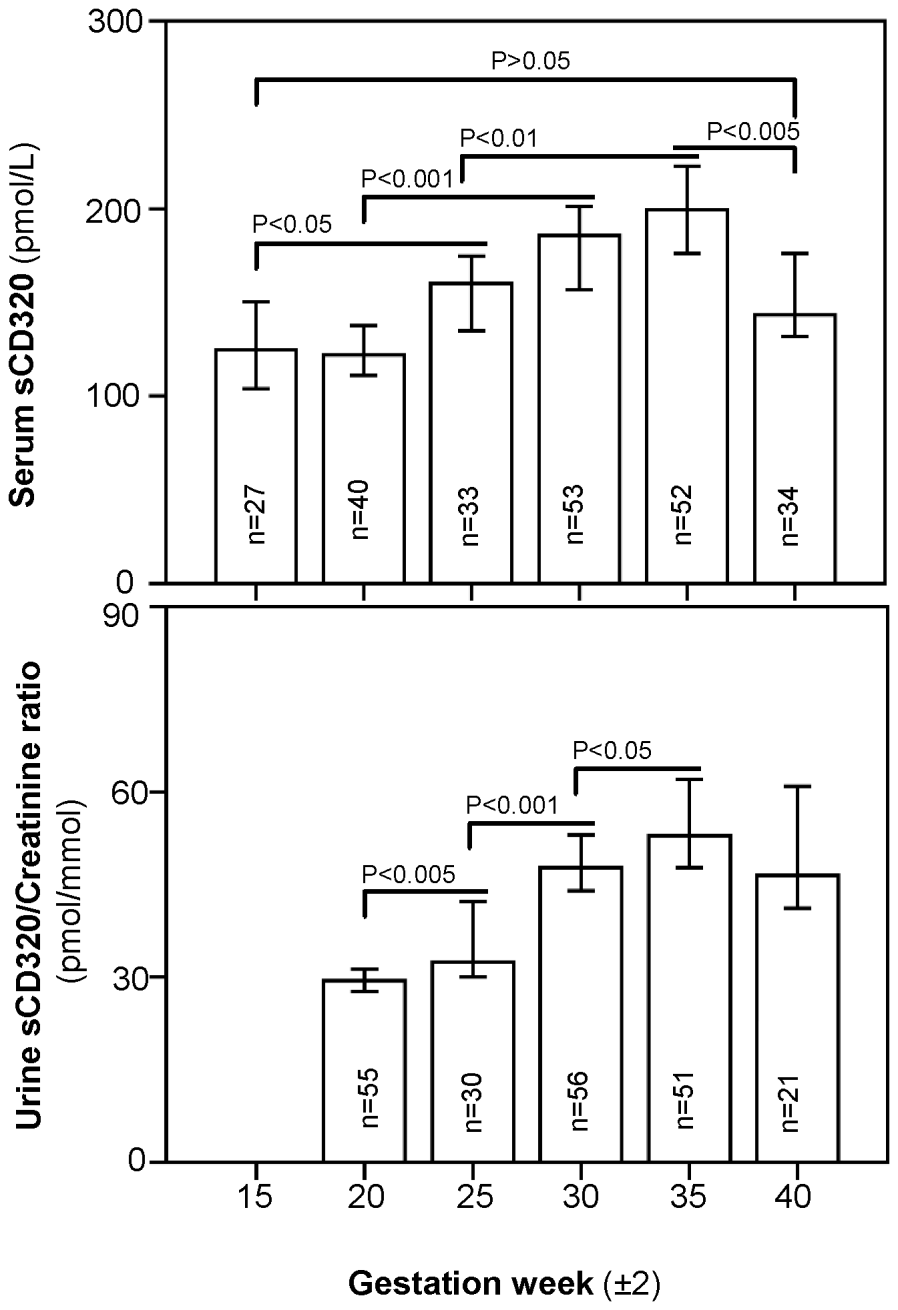
sCD320 in serum and urine during pregnancy. Samples from 21–55 women were analyzed at each gestational period. Median with 95% CI is indicated. P values were calculated employing the Mann–Whitney U test. Urine concentration is expressed relative to creatinine.

The sCD320 concentration during pregnancy in both serum and urine is presented in Figure 1 and its descriptive statistics presented in table 2. The serum sCD320 concentration increased gradually during pregnancy starting from gestation week 20(median =122 pmol/L) and reach a peak at week 35 (median =199 pmol/L) then declined before birth to the median level of 143 pmol/L (week 40).

**Table 2:** Serum and urine sCD320 during pregnancy.

	**Serum sCD320**		**Urine sCD320**	
	(pmol/L)		(pmol/mmol creatinine)	
**Week (±2)**	**n**	**Median(range)**	**n**	**Median(range)**
15	27	125 (87-839)	_	_
20	40	122 (73-746)	55	29 (19-51)
25	33	160 (97-719)	30	32 (21-56)
30	53	185 (94-720)	56	48 (25-88)
35	52	199 (72-672)	51	53 (30-101)
40	34	143 (86-717)	21	47 (30-125)

Descriptive statistics of serum and urine sCD320 at different gestation weeks. Medians (ranges) with number of samples are shown. Abbreviations: sCD320: soluble transcobalamin receptor; n: number of samples in subgroup.

In support to our previous study [11], we found a positive correlation between serum sCD320 and holoTC (Spearman’s rank correlation=0.381, P<0.001, n=199). Also we found a significant correlation between serum sCD320 and total TC (Spearman’s rank correlation=0.412, P<0.001, n=199). Table 3 show descriptive statistics for serum holo- and total TC along with urine total TC during pregnancy.

**Table 3:** HoloTC and total TC during pregnancy.

			**Serum samples**		**Urine samples**	
	**Week(±2)**	**n**	**HoloTC**	**Total TC**	**n**	**Total TC**
			(pmol/L)	(pmol/L)		(pmol/mmol creatinine)
**Median(range)**	15	27	57 (19-190)	840 (615-1265)	_	_
	20	30	79 (50-223)	953 (690-1220)	39	3 (0-13)
	25	33	61 (20-123)	965 (650-1240)	22	3 (1-9)
	30	43	88 (41-173)	1030 (680-1465)	41	2 (0-8)
	35	42	71 (21-152)	1020 (715-1490)	37	1 (0-6)
	40	24	80 (27-209)	1055 (795-1475)	17	3 (0-8)

Descriptive statistics of serum and urine holoTC and urine total TC during pregnancy. Medians (ranges) with number of samples are shown. Abbreviations: n: number of samples in subgroup; TC: transcobalamin.

For the first time we document the presence of sCD320 in urine. We find concentrations that exceed its serum counterpart in all examined gestation weeks (Figure 1, table 2). Urine sCD320 level (expressed as a ratio to urine creatinine) follows serum sCD320 and increase gradually from week 20 (median=29 pmol/mmol) to reach a peak at week 35(median=53 pmol/mmol) (Figure 1, table 2).

**Figure 2:**
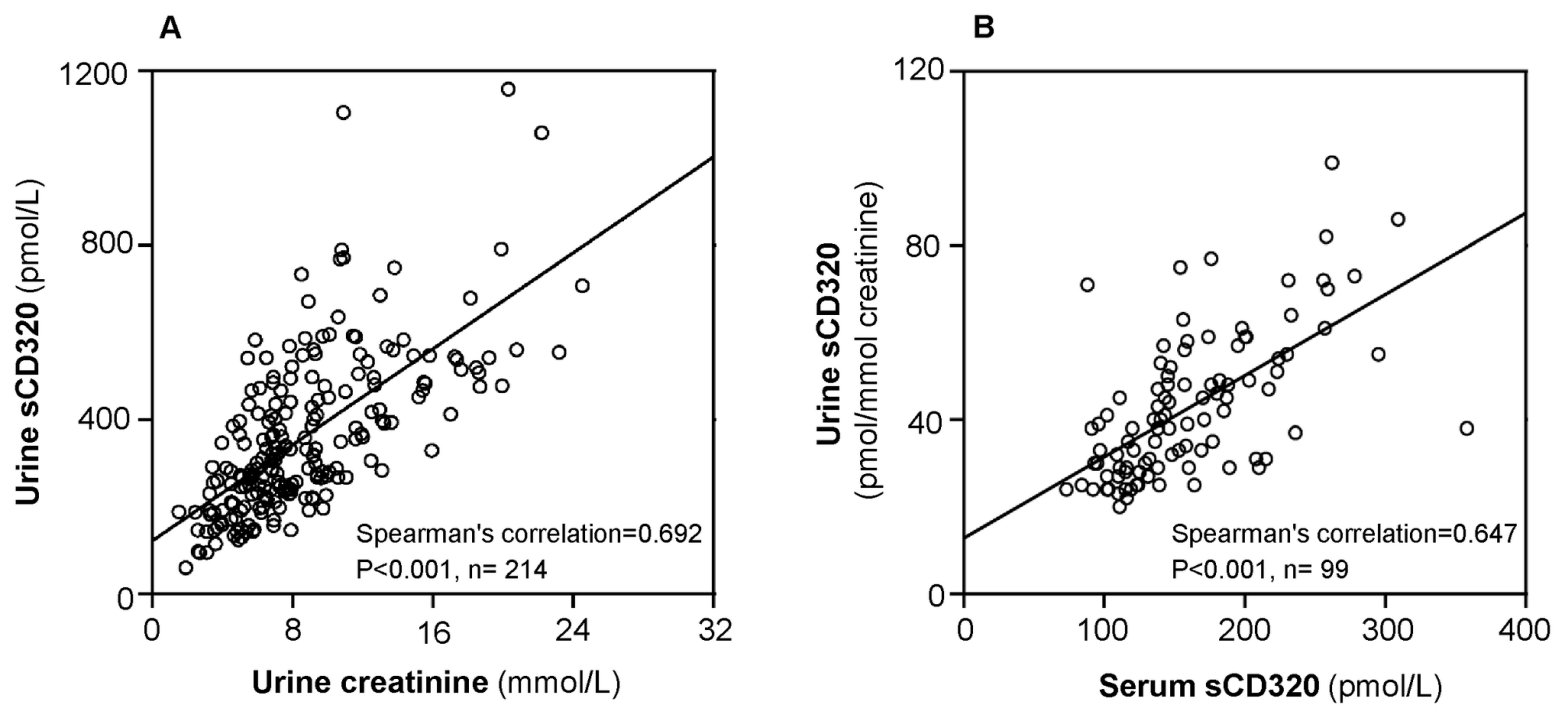
Urinary sCD320 correlates to urinary creatinine and to serum sCD320. The figures are based on results from 40 women in whom samples were collected throughout pregnancy. Correlation of urine sCD320 to urine creatinine (A) and to serum sCD320 (B). P-values obtained using spearman’s correlation.

We observed a strong positive correlation between urine sCD320/creatinine and serum sCD320 levels (Spearman’s correlation=0.647, P<0.001, n=99) and between urine sCD320 to the filtration product, urine creatinine (Spearman’s correlation=0.692, P<0.001, n= 214) (Figure 2) but no correlation between serum total TC and urine total TC/creatinine.

**Figure 3:**
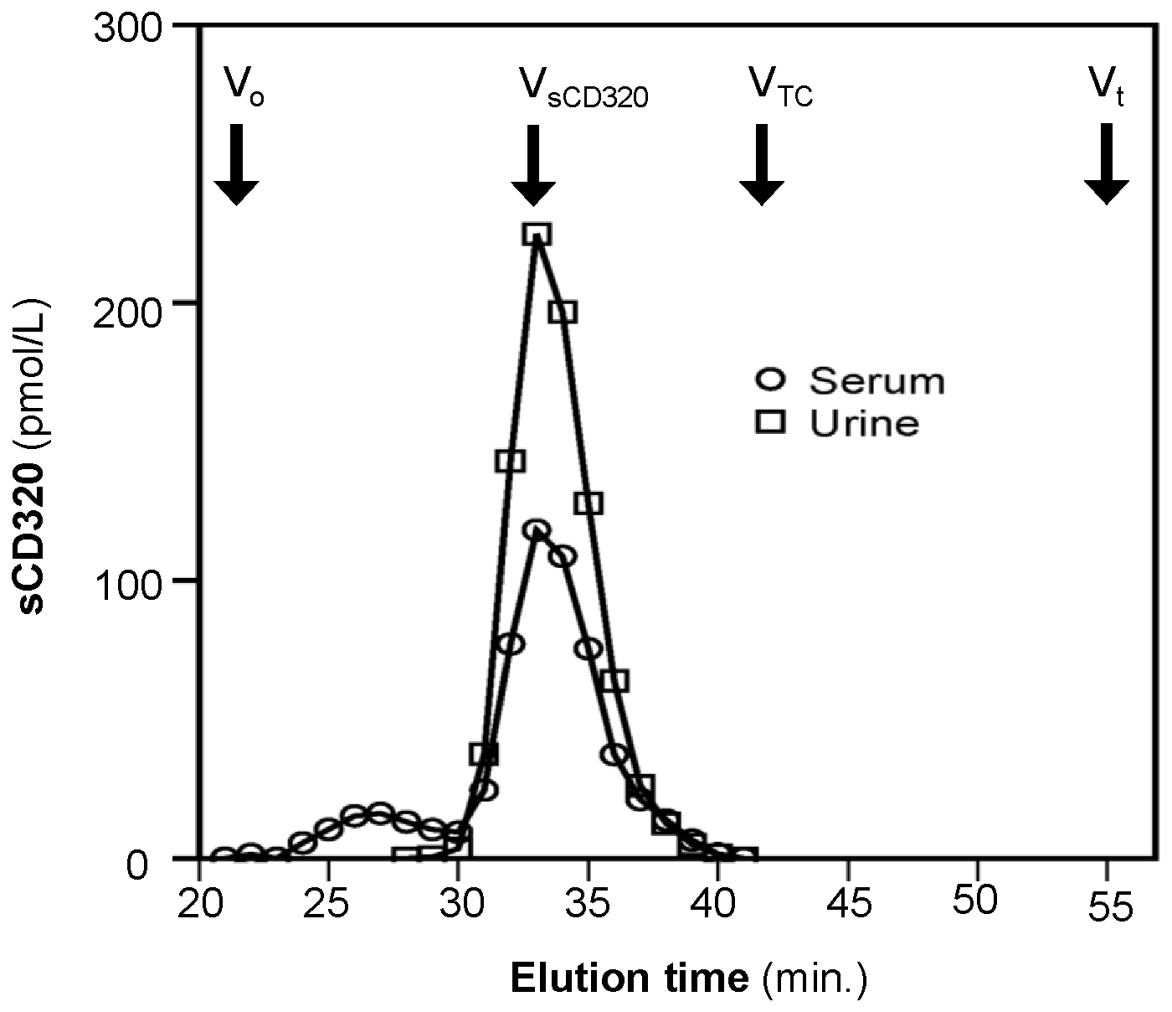
Size exclusion chromatography of sCD320 from pregnant serum and urine samples. Size exclusion chromatography performed on a Superdex 200 column. Arrows indicate elution of void volume (V_o_), donor serum sCD320 (V sCD320), transcobalamin (V_TC_) and total volume (V_t_).

We explored the molecular characteristics of sCD320 in both serum and urine by size exclusion chromatography. sCD320 from serum and urine behaved alike and sCD320 reactivity eluted as a sharp peak with a stokes radius ^≈^ 50 Å, which is identical to the size of sCD320 from donor serum(men and non-pregnant women) [10] (Figure 3). Finally we explored to which degree sCD320 occurred in its free form, and to which degree it was attached to holoTC by analyzing serum samples from 6 women at 15, 25 and 35 weeks of pregnancy before and after precipitation with antibodies against TC. We found comparable fractions of sCD320 to be removed by the precipitation at all-time points studied (Medians % (with 95% CI) are 36(23-47), 36(29-47) and 40(26-53) for weeks 15, 25 and 35 respectively). 

## Discussion

For the first time we report on the molar concentrations of sCD320 in serum and urine, we show increasing levels during pregnancy and surprisingly we report urinary concentrations exceeding those observed in serum. In addition our data suggests that the major part of circulating holoTC is present in complex with its receptor, sCD320.

Early studies showed the human placenta to express a receptor that recognize holoTC [18–20]. More recently the receptor has been purified and characterized from human placenta [9] and realized to be the CD320 molecule. In addition our previous studies have shown a soluble form of CD320 to be present in serum [10,11]. Together these findings paved our way to explore the concentration of sCD320 during the physiological process of pregnancy.

We observed increasing serum values of sCD320 up to gestational week 35 followed by declining values towards birth, and we find it likely that the increase relates to a contribution from placenta where CD320 is expressed abundantly and from which CD320 was purified and characterized [9]. The pattern of release is in accord with the fact that placenta proliferation is diminished during the last weeks of pregnancy [21–23].

We explored the concentration of both TC and sCD320 in urine. As expected very little TC was recovered in the urine. This is in accord with the current view that TC (26 Å) is filtered in the kidney but reabsorbed in the proximal tubules mediated by binding to the multifunctional receptor megalin [5,6]. To our surprise we measured levels of sCD320 in urine that were about two times higher than in serum. This was a totally unexpected result, since the heavily glycosylated sCD320 behaves as a molecule of 50 Å, a size that predict a very limited filtration. The two-pore model of glomerular permeability [24] operates with a large number of small size pores (30-45 Å) and low number of large size pores (110-115 Å), with a calculated ratio of 7 x 10^-7^ for large to small pores based on data from normal rats [25]. The proteins that only pass from the low number large sized pores has a limited fractional plasma-to-urine clearance and as an example IgG2 (55 Å) has a fractional plasma-to-urine clearance of 1.58^-4^. Based on this experimental data it seems unlikely that urinary sCD320 is derived from filtration in the kidney. Never the less our data does point in that direction. First we explored whether we measured a fragment of sCD320 in urine. This was not the case. Serum and urinary sCD320 behaved identically upon gel filtration (Figure 3). Second we observed a strong correlation between urinary sCD320 and the freely filtered waste product creatinine, thirdly serum sCD320 correlated well with the urinary excretion of sCD320 (Figure 2) and finally previous data has shown a relation between serum sCD320 and kidney function as judged from serum creatinine [11]. Together these observations point to an unusual kidney handling of sCD320. Further studies are needed in order to unravel the physiological background and possible implications.

The first assay launched for sCD320 employed a calibrator designed an arbitrary value [10]. Here we benefit from the use of a calibrator prepared from recombinant CD320, allowing us to measure sCD320 in molar concentrations. We report sCD320 to be present in serum in concentrations exceeding that of its ligand, holoTC by a factor of two to three. A previous study has shown that holoTC is recognized by CD320 with a high affinity as compared to apoTC [9]. In addition we have previously shown that part of sCD320 coprecipitates with TC, but since sCD320 could not be measured in a molar unit at the time we were unable to judge to which extent holoTC formed complexes with sCD320. Here we show that around equimolar amounts of holoTC and sCD320 are precipitated by antibodies against TC. The absolute amount precipitated corresponds to the concentration of holoTC and accounts for around one third of sCD320. The results suggest that most of circulating holoTC form complexes with sCD320. The binding is likely to have a relatively low affinity since no complex formation is revealed upon gel filtration of serum (data not shown).

In summary, we report for the first time that serum and urine sCD320 increase during pregnancy and that sCD320 is present in urine in a higher concentration than in serum. The strong correlation between urinary and serum sCD320 and between urine sCD320 and urine creatinine suggests that sCD320 is filtered in the kidney. 
